# Performance Characterization and Optimization of a Miniaturized SERF Atomic Magnetometer via Tunable Laser Power

**DOI:** 10.3390/s26062000

**Published:** 2026-03-23

**Authors:** Peng Shi, Chen Zuo, Qisong Li, Shulin Zhang

**Affiliations:** 1 School of Optical-Electrical and Computer Engineering, University of Shanghai for Science and Technology, Shanghai 200093, China; 233350658@st.usst.edu.cn (P.S.); 243350699@st.usst.edu.cn (C.Z.); liqisong@usst.edu.cn (Q.L.); 2 Superconducting Electronics Laboratory, Shanghai Institute of Microsystem and Information Technology, Chinese Academy of Sciences, Shanghai 200050, China

**Keywords:** atomic magnetometer, Spin-Exchange Relaxation-Free, laser, optical power

## Abstract

**Highlights:**

**What are the main findings?**

- The distinct evolutionary trends of sensitivity, bandwidth, and dynamic range were systematically quantified as a function of tunable laser power in a miniaturized SERF magnetometer.
- An optimized comprehensive performance was achieved within a compact 35 × 22 × 15 mm^3^ probe, featuring 16 fT/√Hz sensitivity, 230 Hz bandwidth, and a ±5.4 nT dynamic range.

**What are the implications of the main findings?**

- Active modulation of laser power enables a flexible transition between ultra-high sensitivity and high-bandwidth operating modes without the need for hardware reconfiguration.
- The established power–performance framework enhances the environmental adaptability and operational robustness of portable biomagnetic sensing systems in diverse application scenarios.

**Abstract:**

Spin-exchange relaxation-free (SERF) atomic magnetometers have emerged as highly promising candidates for ultra-weak magnetic field detection, particularly in biomagnetic imaging, owing to their exceptional sensitivity, amenability to miniaturization, and near-room-temperature operation. While current miniaturized magnetometers typically employ laser chips with fixed optical power, the quantitative impact of laser power on critical performance metrics remains to be fully elucidated. This study systematically investigates the influence of laser power on sensitivity, bandwidth, and dynamic range by incorporating considerations of power broadening, saturation absorption, and noise constraints. A miniaturized probe, integrated with an actively controlled vertical-cavity surface-emitting laser (VCSEL), was developed for experimental validation. Theoretical and experimental results consistently demonstrate that as optical power increases, sensitivity exhibits a non-monotonic dependence, whereas both bandwidth and dynamic range manifest a monotonic upward trend, aligning well with theoretical simulations. The optimized sensor achieved a peak sensitivity of 16 fT/√Hz at 300 μW, while the bandwidth and dynamic range reached 230 Hz and ±5.4 nT at 500 μW, respectively. This work establishes a robust theoretical and experimental framework for the comprehensive performance optimization of laser-integrated miniaturized atomic magnetometers.

## 1. Introduction

Magnetic sensors constitute a pivotal constituent of modern sensing technology, playing an indispensable role in domains such as biomedical engineering, national defense, geophysical exploration, and fundamental scientific research [1,2,3]. In recent years, atomic magnetometry—specifically Optically Pumped Magnetometers (OPMs) operating in the Spin-Exchange Relaxation-Free (SERF) regime—has undergone rapid advancement. The sensitivity of SERF-OPMs has reached the femtotesla (fT/√Hz) level, which is now comparable to that of Superconducting Quantum Interference Devices (SQUIDs) [4,5]. Leveraging their exceptional sensitivity, amenability to miniaturization, and the capacity for near-room-temperature operation [6], SERF-OPMs demonstrate profound potential for ultra-weak magnetic field detection [7]. Notably, the advent of wearable magnetoencephalography (MEG) based on SERF technology has catalyzed a significant research surge in the field of biomagnetism [8]. To date, SERF-OPMs have been successfully deployed in detecting biomagnetic signals, including magnetocardiography (MCG) and MEG [9]. This technology exhibits immense value in clinical applications, such as the early diagnosis of myocardial ischemia and the precise localization of functional brain regions, and is poised to drive the development of next-generation high-end medical imaging modalities [10].

From a fundamental operational perspective, the performance of an atomic magnetometer is primarily governed by core factors including vapor cell fabrication, environmental magnetic noise suppression, and the characteristics of the pump light source. Regarding vapor cell technology, two primary technical paradigms coexist: traditional glass-blowing and Micro-Electro-Mechanical Systems (MEMS) processing. The National Institute of Standards and Technology (NIST) has conducted systematic research into MEMS-based vapor cell fabrication, utilizing silicon-glass bonding to produce millimeter-scale rubidium or cesium cells. Research incorporating these cells has pushed magnetic sensitivity to thresholds better than 25 fT/√Hz [11]. In terms of environmental noise mitigation, achieving high-precision measurements in near-zero fields typically necessitates a combination of passive and active shielding, such as permalloy shields and active compensation coils. Professor M.V. Romalis’s group at Princeton University constructed a gradiometer configuration with a pair of SERF magnetometers and, combined with active field compensation, successfully captured human MCG signals in a standard laboratory environment without the need for large-scale magnetic shielding [12]. These milestones have significantly propelled the practical evolution of SERF-OPM technology.

Concerning the pump laser, early research relied heavily on Ti: sapphire lasers or other bulky light sources. Although these systems provide excellent frequency stability and low noise, their substantial volume, structural complexity, and high power consumption fail to satisfy the stringent requirements for portable magnetometers. Consequently, research has shifted toward miniaturized pump sources, such as vertical-cavity surface-emitting lasers (VCSELs) and distributed feedback (DFB) lasers [13]. Recent efforts have focused on enhancing source stability and modulating laser parameters, including light shift compensation [14] and the homogenization of the pump light field [15]. Tianbo Wu et al. leveraged the power broadening effect in DFB lasers to significantly expand the magnetometer’s bandwidth and dynamic range while maintaining high sensitivity, thereby enabling real-time detection of murine MCG signals [16]. However, in practical engineering applications, contemporary miniaturized SERF-OPMs predominantly utilize VCSEL chips operating at a fixed optical power [17]. Such fixed-parameter operation forces a static trade-off between the sensitivity limit and dynamic response, which restricts the sensor’s adaptability to varying environmental noise levels [7]. While the physical principle of power broadening has been established [18], a systematic investigation into the quantitative ‘performance-tuning envelope’—the dynamic interplay where sensitivity, bandwidth, and dynamic range evolve simultaneously as a function of tunable VCSEL power—remains a significant research gap for integrated probes. Establishing this quantitative mapping is essential for developing next-generation magnetometers capable of transitioning between ultra-high sensitivity (e.g., for stationary MCG) and high-robustness (e.g., for wearable MEG) without hardware reconfiguration.

To address this gap, the present study aims to systematically investigate the influence of laser power on the comprehensive performance of SERF atomic magnetometers by actively modulating the VCSEL driving power. Initially, the physical mechanisms underlying the atomic relaxation process were analyzed theoretically by incorporating the optical pumping rate and power broadening effects. Subsequently, a miniaturized probe was developed for experimental validation, allowing for the characterization of sensor performance under varied optical powers. The results indicate that as the optical power increases, the sensor’s performance metrics exhibit distinct evolutionary trends: sensitivity displays a non-monotonic character, whereas both bandwidth and dynamic range increase significantly. By implementing active power regulation of the laser chip, this work provides an effective dynamic balancing strategy for the holistic performance optimization of miniaturized atomic magnetometers, catering to the distinct sensor requirements across diverse application scenarios.

## 2. Principles and Experimental Configuration

### 2.1. Operating Principles

The experimental measurement system developed in this study is predicated upon the principles of alkali–metal atomic magnetic induction within the spin-exchange relaxation-free (SERF) regime. In this state, the spin-exchange rate significantly outpaces the Larmor precession frequency [19]. In the presence of an external magnetic field, the evolution of atomic angular momentum and polarization can be rigorously characterized via density matrix formalisms. By incorporating both relaxation and optical pumping terms, the spin polarization vector $\vec{P}$ is described by the phenomenological Bloch equation [20,21]:

(1) $$\frac{d\vec{P}}{dt}=D\nabla^{2}\vec{P}+\frac{1}{q}\left(\gamma \vec{P}\times \vec{B}+R_{op}\left(\frac{1}{2}\vec{s}-\vec{P}\right)-R_{rel}\vec{P}\right)$$

where D denotes the diffusion coefficient of the alkali metal atoms ($cm^{2}/s$), quantifying the spatial transport characteristics of the spin polarization vector; γ represents the electron gyromagnetic ratio, which governs the linear correspondence between the Larmor precession frequency and the magnetic field; q is the nuclear slowdown factor, characterizing the buffering effect of nuclear spin on electron spin relaxation; $R_{op}$ and $R_{rel}$ signify the optical pumping rate and the total relaxation rate, respectively; and $\vec{s}$ is the optical pumping vector.

The propagation direction of the laser is defined along the y-axis, designated as the insensitive axis, while the x- and z-axes constitute the two sensitive orthogonal axes. Since the sensor operates within the SERF regime under near-zero magnetic field conditions ($\vec{B}\approx 0$), Equation (1) reduces to the following steady-state equilibrium:

(2) $$\frac{d\vec{P}}{dt}=R_{op}\left(\frac{\vec{s}}{2}-\vec{P}\right)-R_{rel}\vec{P}=0$$

The steady-state polarization $P_{0}$ is subsequently derived as:

(3) $$P_{0}=\frac{R_{op}}{R_{op}+R_{rel}}\cdot \frac{s}{2}$$

As analyzed by Auzinsh and Budker et al. [22], under the assumption that the measurement is governed by shot-noise limited sensitivity, the functional dependence of the total magnetic uncertainty $\delta B_{total}$ on the incident laser power I can be expressed as an analytical equation:

(4) $$\delta B_{total}\propto \sqrt{\delta B_{ph}^{2}+\delta B_{at}^{2}}\propto \sqrt{\frac{a}{I}+b\cdot I}$$

where the term $1/\sqrt{I}$ represents the photon statistics (shot-noise) limit and $\sqrt{I}$ accounts for the pumping-induced relaxation (power broadening). Specifically, the coefficient a represents the photon shot-noise contribution, which is governed by the photodetector’s quantum efficiency and the laser detuning relative to the atomic transition. The coefficient b denotes the atomic back-action noise factor, which characterizes the sensitivity degradation caused by the probe light’s induced stochastic spin-flip events and the resulting power broadening of the magnetic resonance. Equation (4) predicts a characteristic “U-shaped” evolution of sensitivity. In the low-power regime ($I\ll I_{opt}$), δB is dominated by the $1/\sqrt{I}$ shot-noise dependence, where increasing power improves the signal-to-noise ratio of the optical measurement. Conversely, in the high-power regime ($I\gg I_{opt}$), the atomic back-action noise dominates, and the sensitivity deteriorates as $\sqrt{I}$ due to the pronounced power broadening effect. This theoretical framework establishes a deterministic global optimum ($I_{opt}$), where these competing noise sources reach a physical balance.

Based on the Bloch formalism in Equation (1), the effective relaxation rate 1/τ can be expressed as [18]:

(5) $$\frac{1}{\tau }=\frac{1}{T_{p}}+\frac{1}{T}=R_{op}+R_{rel}$$

where τ is the effective relaxation time constant, resulting from the synergistic effects of the spin relaxation time T and the optical pumping time constant $T_{p}$. The intrinsic relationship between τ and the sensor’s response speed reflects the temporal agility of the system in recovering equilibrium following a perturbation. The bandwidth (BW) represents the frequency spectrum over which the sensor maintains an effective response, typically defined as the frequency interval where the output signal power diminishes to −3 dB of its maximum value. Based on the first-order relaxation model [23], it can be formulated as:

(6) $$BW=\frac{1}{2\pi \tau \;}=\frac{1}{2\pi }\;\left(R_{op}+R_{rel}\right)$$

Furthermore, when a modulated magnetic field is applied and the output from the photodetector (PD) is demodulated via a lock-in amplifier, the resulting demodulated output voltage signal, denoted as $V(B_{z})$ (measured in Volts), is given by [23]:

(7) $$V\left(B_{z}\right)=A_{0}\frac{\gamma B_{z}\tau }{1+{\left(\gamma B_{z}\tau \right)}^{2}}$$

where $A_{0}$ is the signal amplitude factor. As shown in Equation (7), $A_{0}$ is governed by $A_{0}\propto R_{op}\cdot P_{0}$, indicating a proportional relationship with both the optical pumping rate $R_{op}$ and the steady-state polarization $P_{0}$. This response function exhibits a characteristic dispersive Lorentzian lineshape. The dynamic range—the magnetic field interval over which the system maintains a maximal linear response—is dictated by the quasi-linear regime of this function. Specifically, the upper limit of the dynamic range is proportional to the relaxation rate 1/τ, which increases with laser power.

### 2.2. Experimental Configuration

**Figure 1:**
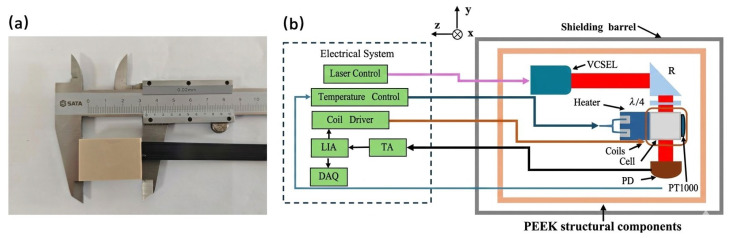
Schematic representation of the magnetometer experimental configuration. (**a**) Physical prototype of the magnetometer probe with external dimensions of $35\times 22\times 15{\;\mathrm{mm}}^{3}$; (**b**) systematic block diagram of the magnetometer architecture, PEEK (Polyether Ether Ketone) structural components of the sensor.

Contemporary miniaturized SERF magnetometers predominantly utilize vertical-cavity surface-emitting lasers (VCSELs) as pump sources, while distributed feedback (DFB) lasers have also seen limited research applications. VCSELs have become the mainstream choice due to their compact footprint, low power consumption, and operational simplicity. However, commercial VCSELs (such as those from Vixar, Plymouth, MN, USA) typically exhibit an output power of approximately 100 μW; sustained operation at higher power levels can significantly curtail device longevity, thereby constraining the long-term reliability of the system. To address this limitation, we implemented a domestically developed laser chip through customized packaging to fabricate a miniaturized pump source optimized for single-beam SERF magnetometers. The physical probe, with compact dimensions of $35\times 22\times 15{\;\mathrm{mm}}^{3}$, is depicted in Figure 1a.

The magnetometer probe is housed within a five-layer permalloy magnetic shielding cylinder, which ensures a residual magnetic field of approximately 2 nT. The systematic operation of the sensor follows the architecture illustrated in the block diagram in Figure 1b. Initially, the VCSEL is driven by high-precision current and temperature control modules to emit a narrow-linewidth laser at 795 nm, resonant with the ^87^Rb D_1_ transition. The beam is collimated, redirected via a right-angle mirror, and passed through a λ/4 waveplate to generate circularly polarized light. This beam is then incident upon a $4\times 4\times 4{\;\mathrm{mm}}^{3}$ rubidium vapor cell containing ^87^Rb droplets and 600 Torr of $N_{2}$ buffer gas, the latter serving to quench excited states and mitigate collisional relaxation.

To sustain the SERF regime, several critical measures are integrated: first, a non-magnetic heating element driven by a 300 kHz AC signal, in conjunction with a PT1000 thermistor, stabilizes the cell temperature at 150 °C (with a precision of ±0.04 °C). Second, a three-axis Helmholtz coil fabricated on a flexible printed circuit (FPC) is utilized to compensate for the residual magnetic field, effectively nulling the field to within 0.1 nT to ensure that the static residual environment does not consume or restrict the ±5.4 nT dynamic range of the magnetometer. In this single-beam architecture, the circularly polarized light facilitates both optical pumping and signal interrogation. Under SERF conditions, the target magnetic field components perpendicular to the optical axis induce Larmor precession of the atomic spins, which subsequently modulates the polarization or intensity of the transmitted light through magneto-optical effects (e.g., Faraday rotation or absorption changes). An 800 Hz sinusoidal modulation is applied to the z-axis coil, and the resulting transmitted light is captured by a photodiode (PD). The signal is converted via a transimpedance amplifier (TA) and demodulated by a lock-in amplifier (LIA). Finally, the digitized output is recorded by a data acquisition (DAQ) system to achieve high-precision magnetic field measurement.

## 3. Experimental Results and Analysis

### 3.1. Characterization of VCSEL Performance

**Figure 2:**
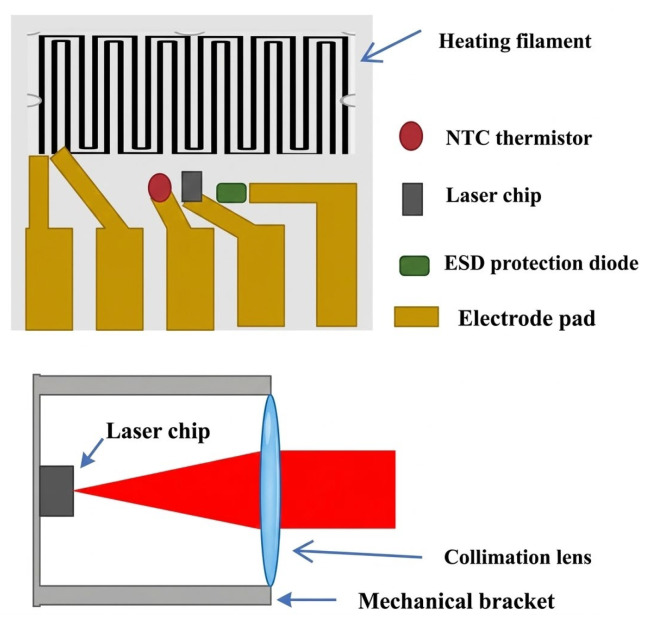
Laser structure and beam collimation (NTC: Negative Temperature Coefficient; ESD: Electrostatic Discharge).

In the context of SERF atomic magnetometers, the pump light source must satisfy stringent requirements, specifically regarding its non-magnetic properties and the high-fidelity stability of both its frequency and output power. Currently, the most prevalent light sources utilized in this field include vertical-cavity surface-emitting lasers (VCSELs) and distributed feedback (DFB) lasers [13]. Owing to its inherent advantages—namely low power consumption, a minimal spatial footprint, and cost-efficiency—the VCSEL has emerged as the predominant candidate for commercial-grade atomic magnetometry systems [11]. In this study, a VCSEL with a central emission wavelength of 795 nm was employed, precisely tailored to resonate with the $D_{1}$ line transition of ^87^Rb. The internal architecture and optical configuration of the VCSEL source are detailed in Figure 2. The upper panel illustrates the layout of the laser package, featuring a resistive heater with an interleaved serpentine pattern designed to suppress parasitic magnetic fields while maintaining thermal stability. Below the heater, five electrode pads are arranged for the heating element and an NTC thermistor for real-time temperature monitoring. The system also integrates a 795 nm laser chip and an ESD protection diode. The lower panel shows the cross-sectional view of the collimation module. A lens transforms the divergent beam from the laser chip (indicated by the black rectangle) into a parallel light beam. As shown in the systematic diagram (Figure 1b), this collimated light is redirected by a right-angle prism and circularly polarized via a λ/4 waveplate before entering the vapor cell. In this single-beam architecture, the light serves both as a pump and a probe, with the transmitted signal eventually captured by a photodetector (PD) for subsequent electronic processing and analysis.

**Figure 3:**
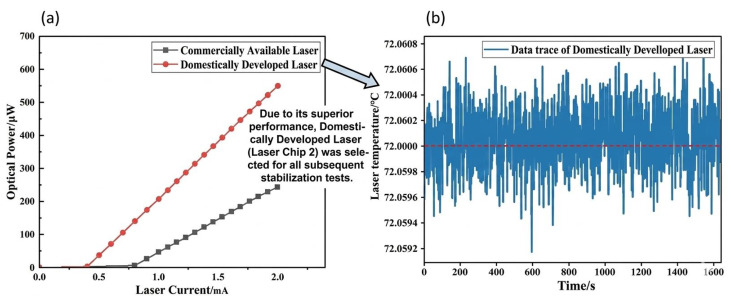
Characterization and selection of laser performance. (**a**) Power–current (P-I) characteristics of the standard commercial VCSEL (Laser 1) and the high-efficiency domestically developed VCSEL (Laser 2); (**b**) temperature control stability of the selected Laser 2, where the red dashed line represents the target temperature value for laser control; this laser was utilized for all subsequent magnetometer performance evaluations.

Two distinct laser chip variants—a standard commercial VCSEL (Laser 1) and a high-efficiency domestically developed VCSEL (Laser 2)—were encapsulated and characterized in this study to determine the optimal pump source for the miniaturized probe. The optical power output was regulated by modulating the drive current and quantitatively assessed using a PM100D (Thorlabs, Inc., Newton, NJ, USA) power meter. As illustrated in Figure 3a, the results demonstrate that the domestically developed laser (Laser 2) possesses a threshold current of 0.39 mA, which is markedly lower than the 0.77 mA recorded for Laser 1. At a reference drive current of 2 mA, Laser 2 achieves an output of approximately 600 μW, substantially surpassing Laser 1 in terms of electro-optical conversion efficiency. Although the subsequent magnetometer characterization primarily utilizes the 200–500 μW range (corresponding to approximately 1.1–1.7 mA for Laser 2), this comparison at 2 mA provides a clear quantification of the superior slope efficiency of the domestic chip. Higher efficiency ensures lower thermal dissipation at any given optical power, which is critical for maintaining the sub-millikelvin thermal stability required in miniaturized SERF sensors. Owing to its lower power consumption and reduced thermal load, Laser 2 was selected as the exclusive light source for all subsequent performance characterizations in this manuscript.

To ensure wavelength stability and precise alignment with the ^87^Rb $D_{1}$, resonance line, the laser temperature ($T_{set}$) and drive current (I) were subjected to synergistic regulation. The experimental protocol prioritized setting the target optical power first, followed by the implementation of a PID-based closed-loop control algorithm to stabilize the emission wavelength at 795 nm. As depicted in Figure 3b, the temperature stability was maintained with an exceptional precision of within ±0.001 °C, Given that the typical frequency-to-temperature tuning coefficient for a VCSEL is approximately 30 GHz/°C, this high thermal precision translates to a frequency stability of about ±30 MHz. Since the absorption linewidth of ^87^Rb is broadened to the GHz range by the 600 Torr $N_{2}$ buffer gas, a fluctuation of 30 MHz is negligible, ensuring that the laser remains robustly on-resonance. This guarantees a high-fidelity and stable spectral output for the sensor system.

### 3.2. Characterization of Sensor Sensitivity

**Figure 4:**
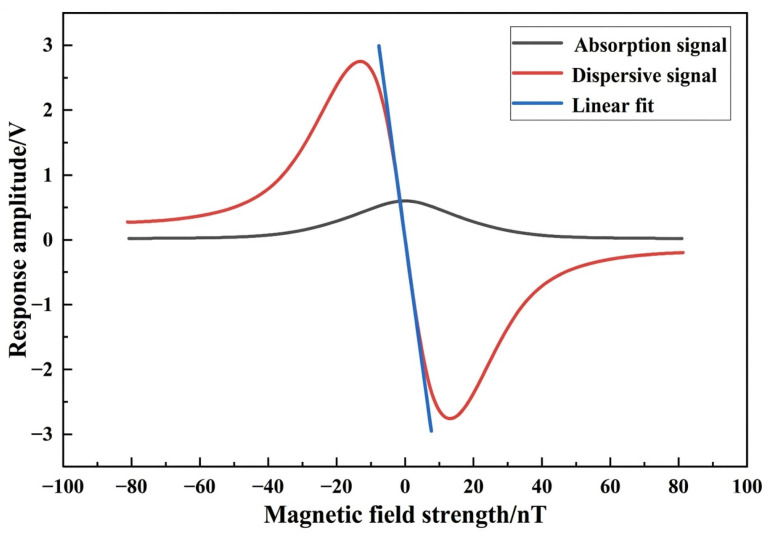
Measured magnetic resonance spectrum at 300 μW. The black curve (absorption signal) and the red curve (demodulated dispersive signal) demonstrate the system’s alignment with the theoretical model in Equation (7).

To rigorously assess the fundamental performance metrics of the miniaturized atomic magnetometer, we performed a systematic characterization of its sensitivity limit and noise floor. The sensor was housed within a five-layer permalloy magnetic shielding cylinder, with the vapor cell temperature stabilized at 150 °C (maintaining a control precision of ±0.04 °C). With the laser power optimized at approximately 300 μW, the magnetic resonance absorption signal and dispersive signal were acquired by scanning the transverse DC magnetic field, as illustrated in Figure 4. The absorption signal represents the DC intensity change in the light transmitted through the atomic ensemble, reflecting the optical resonance dip. In contrast, the dispersive signal is obtained by demodulating the output from a photodetector via a lock-in amplifier, which represents the experimental realization of the theoretical response $V(B_{z})$ described in Equation (7).

A subsequent fitting analysis yielded a magnetic linewidth of ΔB≈36 nT (corresponding to a frequency linewidth of Δν≈1 kHz). As established in Equation (5), this magnetic linewidth is intrinsically determined by the effective relaxation rate 1/τ, where a narrower linewidth indicates a longer relaxation time. This measured linewidth is significantly narrower than the estimated spin-exchange rate ($R_{SE}\approx 110\;\mathrm{kHz}$), thereby confirming that the system has successfully attained the prerequisites for operation within the SERF regime. As illustrated in Figure 4, the magnetic-to-voltage scale factor ($V_{B}$) was determined to be 0.387 V/nT by performing a linear fit on the dispersive signal around the zero magnetic field. This scale factor is critical for converting technical noise into magnetic equivalent units. To suppress 1/f noise and circumvent interference from technical characteristic peaks, a sinusoidal modulation frequency of 800 Hz was implemented for signal extraction.

**Figure 5:**
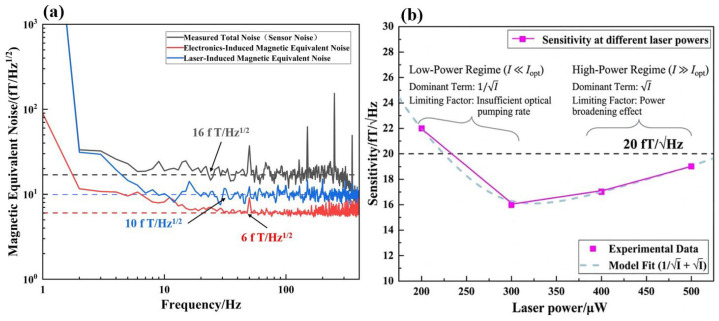
Quantitative characterization of magnetometer sensitivity and noise contributions. (**a**) Total magnetic noise floor (16 fT/√Hz) and the magnetic equivalent noise contributions from laser intensity (10 fT/√Hz) and electronic sources (6 fT/√Hz). The residual noise includes the theoretically estimated spin-projection noise (~3.5 fT/√Hz) and environmental fluctuations; (**b**) non-monotonic evolution of sensitivity as a function of incident laser power, fitted with the analytical model derived from the trade-off between atomic polarization and power broadening.

Four discrete experimental data points were established within the laser power range of 200–500 μW, with the system demonstrating an optimal sensitivity of 16 fT/√Hz. To facilitate a direct comparison of various noise sources, the raw voltage noise spectra of the laser and electronics were converted into magnetic equivalent noise by dividing them by the measured scale factor ($V_{B}=0.387$ V/nT, derived from Figure 4), as depicted in Figure 5a. The reported sensitivity values in Figure 5b were extracted by averaging the noise floor amplitude in the stable 30–50 Hz frequency band, where the noise is free from 1/f interference. A comprehensive noise analysis reveals that the sensor’s noise floor is primarily dominated by the synergistic contributions of spin-projection noise, laser intensity noise, and residual technical noise.

Furthermore, the influence of optical power on magnetic sensitivity was systematically scrutinized within the 200–500 μW interval. The results manifest a distinct non-monotonic dependence of sensitivity on laser power (see Figure 5b): the sensitivity was recorded at 22 fT/√Hz at 200 μW, achieved its peak performance of 16 fT/√Hz at 300 μW, and subsequently deteriorated to 17 fT/√Hz and 19 fT/√Hz as the power further increased to 400 μW and 500 μW, respectively. To verify the physical nature of this observed optimum, the experimental data in Figure 5b were fitted using the analytical model derived in Equation (4). As shown by the dashed blue curve, the measured points exhibit excellent agreement with the theoretical prediction ($R^{2}>0.98$), confirming that the 300 μW minimum is a deterministic physical result of the competition between photon shot noise and power broadening, rather than a stochastic measurement artifact.

This investigated power range (200–500 μW) corresponds to a transition in the atomic saturation parameter ($S=R_{op}/R_{rel}$) from approximately 0.8 to 3.0. In the low-power regime ($I\ll I_{opt}$), the sensitivity is dominated by the $1/\sqrt{I}$ term, where the performance is restricted by an insufficient optical pumping rate, which limits the steady-state polarization degree. Conversely, at higher power levels ($I\gg I_{opt}$), the sensitivity follows a $\sqrt{I}$ dependence because the pronounced power broadening effect curtails the transverse relaxation time, thereby leading to the observed degradation in magnetic sensitivity.

Experimental evidence demonstrates that maintaining the laser power within the 300–500 μW range ensures that the magnetic sensitivity remains consistently below the 20 fT/√Hz threshold. This establishes an optimal operational window that successfully reconciles high detection sensitivity with enhanced system robustness across varying saturation levels. Consequently, such a configuration is highly conducive to sustaining stable, long-term performance even when deployed in complex and challenging environmental conditions.

### 3.3. Sensor Bandwidth and Dynamic Range

The dynamic performance of a magnetometer is a pivotal determinant of its practical utility. This study systematically investigates the dependence of sensor bandwidth and dynamic range on incident laser power, the underlying physical mechanisms of which are elucidated through the relaxation processes within the Bloch equations.

**Figure 6:**
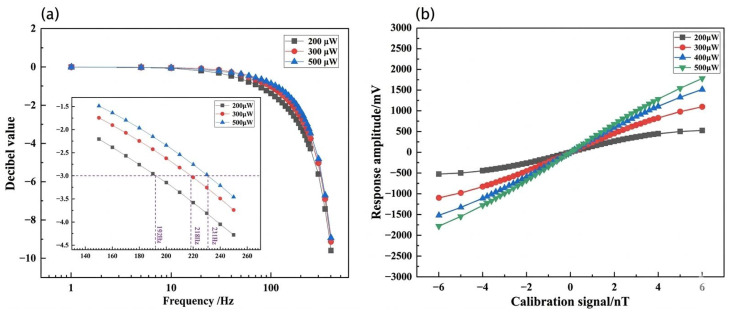
Dynamic response characteristics of the sensor. (**a**) Frequency bandwidth as a function of incident laser power; (**b**) evolution of the dynamic range under varying laser power levels.

Bandwidth characterizes the magnetometer’s capability to track transient fluctuations in the magnetic field. Experimental observations reveal that the −3 dB bandwidth exhibits a linear expansion with increasing laser power, which is in excellent agreement with the theoretical prediction in Equation (6) (Figure 6a). This linear trend is characterized across the power range of 200–500 μW, where the optical pumping rate $R_{op}$ transitions from the under-saturated regime to the saturated regime ($R_{op}\approx 0.8R_{rel}$ to $3R_{rel}$). Specifically, at a power level of 500 μW, the bandwidth reaches 1–231 Hz. This phenomenon is attributed to the enhancement of the optical pumping rate, $R_{op}$. In the Spin-Exchange Relaxation-Free (SERF) regime, the system bandwidth is primarily governed by the effective relaxation time, τ. Increasing the laser power augments $R_{op}$, thereby shortening τ and concomitantly broadening the system’s spectral response range.

The dynamic range defines the maximum magnetic field interval within which the sensor maintains a linear response. To ensure measurement fidelity in practical biomagnetic sensing, we defined the dynamic range using a conservative linearity error threshold of <5%. This specific threshold was chosen to maintain the sensor within its quasi-linear regime while providing a consistent metric for comparing the performance envelope across different laser power levels. The results indicate that the dynamic range is also robustly correlated with laser power (Figure 6b). The dynamic range, measured at ±2.4 nT for 200 μW, extends to ±5.4 nT as the power increases to 500 μW. The expansion of the dynamic range serves as experimental evidence that elevating laser power enhances the resilience of atomic spins against magnetic saturation, satisfying the relationship $B_{sat}\propto 1/\tau$. Consequently, while peak sensitivity is achieved at 300 μW, the 500 μW configuration represents the optimized operating point for high-dynamic applications, providing a superior dynamic range and bandwidth while maintaining a sensitivity better than 20 fT/√Hz.

From an engineering perspective, the transition to the 500 μW mode is critical for practical applications in unshielded or weakly shielded environments. The expansion of the dynamic range to ±5.4 nT significantly enhances the sensor’s resilience against signal saturation caused by residual Earth’s magnetic fields or motion artifacts during wearable biomagnetic measurements. Furthermore, the elevated bandwidth of 231 Hz ensures a flat frequency response across the 50/60 Hz power-line interference spectrum and its lower-order harmonics. This characteristic is vital for multi-channel gradiometer configurations, as it reduces phase-lag disparities between sensors, thereby improving the Common-Mode Rejection Ratio (CMRR) and enabling more effective rejection of environmental technical noise.

## 4. Conclusions

This research systematically investigated the influence of laser chip power on the performance of a Spin-Exchange Relaxation-Free (SERF) atomic magnetometer through a combination of analytical modeling and experimental characterization. By developing a miniaturized sensor probe, the impact of incident laser power on sensitivity, frequency bandwidth, and dynamic range was comprehensively evaluated. The experimental results were validated against a theoretical framework derived from the quantum noise limit, confirming that the sensitivity exhibits a deterministic U-shaped dependence on laser power due to the trade-off between atomic polarization and power broadening.

The study identifies two distinct optimized operating windows: a peak sensitivity of 16 fT/√Hz is achieved at 300 μW, while an optimized comprehensive performance—balancing a 231 Hz bandwidth and a ±5.4 nT dynamic range with a 19 fT/√Hz sensitivity—is reached at 500 μW. Based on the established quantitative relationship between power and performance, the sensor can flexibly transition between ultra-high sensitivity and high-bandwidth/robustness operating modes without hardware modifications. This “gear-shift” capability significantly enhances its environmental adaptability and operational robustness in diverse application scenarios. Specifically, the high-sensitivity mode is optimized for stationary, high-fidelity signals like fetal magnetocardiography, while the high-robustness mode provides the necessary dynamic headroom to prevent saturation and maintain high CMRR amidst the complex interference and motion artifacts typical of wearable MEG systems. Future work will focus on addressing the thermal stability of the atomic system, further increasing the integration level of the miniaturized probe, and implementing closed-loop power regulation to further augment the overall sensor performance.

## Data Availability

The original contributions presented in this study are included in the article. Further inquiries can be directed to the corresponding author.

## Abbreviations

The following abbreviations are used in this manuscript:

SERF	Spin-exchange relaxation-free
VCSEL	vertical-cavity surface-emitting laser
OPMs	Optically Pumped Magnetometers
SQUIDs	Superconducting Quantum Interference Devices
MCG	magnetocardiography
MEMS	Micro-Electro-Mechanical Systems
NIST	National Institute of Standards and Technology
MEG	magnetoencephalography
DFB	distributed feedback
VCSELs	vertical-cavity surface-emitting lasers
PD	photodetector
FPC	flexible printed circuit
TA	transimpedance amplifier
LIA	lock-in amplifier
DAQ	data acquisition
